# Analysis of Apoptosis in Cultured Human Vitrified Ovarian Tissue in the Presence of Leukemia Inhibitory Factor

**Published:** 2018

**Authors:** Maasoume Abdollahi, Mojdeh Salehnia, Saghar Salehpour, Shahram Pour-Beiranvand

**Affiliations:** 1- Department of Anatomical Sciences, Medical Sciences Faculty, Tarbiat Modares University, Tehran, Iran; 2- Infertility and Reproductive Health Research Center (IRHRC), Shahid Beheshti University of Medical Sciences, Tehran, Iran

**Keywords:** Apoptosis, Caspase-3/7, *In vitro* culture, Leukemia inhibitory factor, Ovarian tissue, Vitrification

## Abstract

**Background:**

For improving the human ovarian tissue culture, this study was designed to assess the incidence of apoptosis in this tissue following vitrification and *in vitro* culture in the presence of leukemia inhibitory factor (LIF) as an anti-apoptotic factor.

**Methods:**

After collecting the ovarian tissue samples they were divided into non-vitrified and vitrified groups and cultured for 14 days in the presence and absence of LIF then morphological, ultrastructural and steroidogenesis studies, TUNEL and caspase-3/7 assays, and apoptosis analysis by real time RT-PCR were done in all groups. The data were analyzed by independent t-tests and the real time RT-PCR results were compared by one-way ANOVA (p-values of <0.05 were considered significant).

**Results:**

No significant difference was observed between non-vitrified and vitrified groups in normality rate of follicles, the levels of hormones, TUNEL positive cells and caspase-3/7 activity. But in all LIF-treated groups, the levels of 17-β estradiol and progesterone were higher and TUNEL signals and caspase-3/7 activity were lower than non-LIF treated groups. The expression of Fas and FasL genes was higher in vitrified group in comparison with non-vitrified group but the expression of other genes was not significantly different. In LIF-treated groups, the expression of pro-apoptotic genes was significantly lower and the expression of anti-apoptotic genes was higher than non-LIF treated group.

**Conclusion:**

The vitrification of human ovarian tissue did not increase the incidence of apoptosis at the morphological and molecular levels during long term culture and LIF improves the survival and development of cultured follicles.

## Introduction

An alternative technique for fertility preservation in young women is the cryopreservation of ovarian tissue by vitrification and an increased attention has been focused recently on this technique. Vitrification–a process of solidification without ice crystallization–provides better preservation for ovarian tissue with respect to follicular integrity and function (1–5).

*In vitro* culture of human ovarian tissue following cryopreservation is recommended to allow follicular development. The viability of cultured human ovarian tissue is improved by the addition of various growth factors to the culture media (6–10).

Leukemia inhibitory factor (LIF) is a glycoprotein that belongs to the interleukin-6 family; it is involved in various biological activities, including cell growth and differentiation (11). LIF has a critical role as a survival factor in several cell types during *in vitro* culture, acting via phosphoinositide 3-kinase pathways (12, 13). LIF induces the expression of anti-apoptotic molecules such as Bcl-2 and also plays an essential role in protective mechanisms against oxidative injury during induced cell apoptosis (14). Moreover, its anti-apoptotic effects on several cell types, such as myocytes, oligodendrocytes and mouse embryonic stem cells, have been demonstrated (13, 15–19). It has been reported that LIF is important for the promotion of *in vitro* folliculogenesis in the mouse (20, 21), and it may improve *in vitro* growth and maturation of murine vitrified preantral follicles (21). However, to the authors’ knowledge, there has been no report describing supplementation of culture media with LIF to provide enhancement of human *in vitro* follicular growth as a consequence of its anti-apoptotic effect.

Controversial reports have been published regarding the occurrence of apoptotic cell death immediately after warming of vitrified human ovarian tissue (22–25). It has been shown that vitrification and ultrarapid protocols did not affect the incidence of apoptosis in human ovarian tissue after warming and 24 *hr* of *in vitro* culture (25), but at the molecular level, there were some slight differences in the level of expression of apoptosis-related genes between the vitrified and non-vitrified groups (26). However, apoptosis is a dynamic process and it may need more time to affect the long-term survival of tissue or follicular development. There have been no reports in the literature describing an analysis of the incidence of apoptosis during long-term culture of vitrified human ovarian tissue.

Thus, the objective of this study was to investigate firstly the effect of vitrification, and secondly LIF supplementation as an anti-apoptotic and survival factor on the follicular development and incidence of apoptosis after long-term culture of human ovarian tissue. The assessments were done at the morphological, ultrastructural, biochemical, and molecular levels.

## Methods

### Chemicals:

Unless mentioned otherwise, all reagents and chemicals used in the present study were purchased from Sigma-Aldrich (St, Louis, USA).

### Human ovarian tissue:

Human ovarian tissue was obtained from 25 healthy women aged between 24 and 35 years after caesarean section. The women gave their consent, and the procedure was approved by the Ethics Committee of Tarbiat Modares University (Ref. No. 5274856). Ovarian biopsy specimens approximately 5×5×1 *mm*^3^ were placed in tubes containing pre-warmed and equilibrated Leibovitz L-15 medium with 10 *mg/ml* human serum albumin (HSA; Biotest, Germany), 100 *IU/ml* penicillin and 100 *mg/ml* streptomycin; the samples were transferred to the laboratory within 1–2 *hr* on ice. The ovarian cortexes were cut into small pieces (2.5×1×1 *mm*^3^) under a sterile laminar air flow in Leibovitz L-15 medium.

### Experimental design:

After preparation of the ovarian biopsy specimens, they were cut into several fragments then these tissue fragments were divided randomly in both non-vitrified and vitrified groups. After warming the vitrified samples, the tissue fragments were cultured in the presence or absence of LIF in both groups (Vitrified and non-vitrified groups). The vitrified and non-vitrified human ovarian tissues were subjected to *in vitro* culture for 14 days then the assessment of apoptosis was done by several techniques. Thus, there were four groups including non-vitrified–LIF^−^, vitrified–LIF^−^, vitrified–LIF^+^ and non-vitrified–LIF^+^ groups. To compare the same tissue under different conditions, fragments of each biopsy specimen were included in almost all experimental groups. The apoptosis induced mice thymic tissue and apoptosis induced human ovarian tissue were used in this study as technical controls.

### Vitrification and warming procedures:

The vitrification procedure was based on a method used previously (27), with some modifications. The vitrification solution was EFS40% containing 40% ethylene glycol (EG; *v/v*), 30% ficoll 70 (*w/v*), and 0.5 *M* sucrose supplemented with 0.21% HSA. Ovarian cortical pieces were dehydrated in the vitrification solution for 5 *min*, with three changes. Subsequently, the tissues were loaded into cryovials with a minimal amount of vitrification solution, exposed to nitrogen vapor phase for 30 seconds and finally plunged into liquid nitrogen and stored for one month.

The cryovials containing vitrified tissues were warmed at room temperature for 20 seconds and then placed in a 37*°C* water bath until melted. The warmed tissues were rinsed in solutions of 1, 0.5 and 0.25 *M* sucrose, respectively, at room temperature for 5 *min*. They were equilibrated in Mc Coy’s culture medium for 30 *min* before any assessment.

### Long-term tissue culture conditions:

The tissues were cultured (n=45 fragments for each group) separately on inserts (0.4 *μm* Millicell Culture Plate Inserts, Millipore, France) in six-well culture dishes containing 2 *ml* of McCoy’s culture medium (pH=7.2–7.4) supplemented with 1.25% HSA, 1% insulin–transferrin–selenium (ITS), 100 *IU/ml* penicillin, 100 *mg/ml* streptomycin, 2 *mM* glutamine, 0.3 *IU/ml* human recombinant follicle-stimulating hormone (rFSH or Gonal-f; Serono, Switzerland), and 50 *g/ml* ascorbic acid at 37*°C* in an atmosphere of 5% CO_2_ in air for 14 days (10).

In the LIF treated groups, 50 *ng/ml* recombinant human LIF was added to the culture medium (20). Subsequently, 500 *μl* of medium was collected from each well in all groups every 2 days and replaced with fresh medium. The media collected at 2 and 14 days of cultivation were stored at −20*°C* for hormonal assay.

### Apoptosis induction in tissue:

For preparation of technical positive control groups, the apoptosis was induced in the thymus tissue of 3-week-old NMRI (National Medical Research Institute) mice by giving an intraperitoneal injection of 10 *mg/kg* of dexamethasone (25). Approval for this study was obtained from the Animal Research Ethical Committee of Tarbiat Modares University. After 16 *hr*, the mice (n=9) were sacrificed by cervical dislocation; their thymus tissue was dissected and stored at −80*°C* for caspase-3/7 and TUNEL assays.

### Apoptosis induction in human ovarian tissue:

Fresh human ovarian tissue samples (n=3) were cultured in media supplemented with 0.5 *mg/ml* actinomycin D for 19 *hr* to induce apoptosis (28), as a control for DNA laddering assessment. The tissues were stored at −80*°C* until assessment.

### Light microscopy (LM):

The human ovarian tissue samples (n=5 in each subgroup) were fixed in 4% formaldehyde and paraffin, serially cut into 5 μm sections, and every 10th section was stained with hematoxylin and eosin and assessed under a light microscope. The developmental stages of the follicles were classified by the shape and number of layers of granulosa cells (25).

### Hormonal assay:

The concentrations of 17-β estradiol, progesterone and DHEA (Dehydroepiandrostrone) were analyzed using kits (Monobind, USA; DiaPlus, USA; Monobind, USA, respectively) (n=5 for each hormonal assessment at day 2 or 14 for each subgroup).

### Electron microscopy:

The tissue samples (n=5 in each subgroup) were fixed in 2.5% glutaraldehyde in phosphate-buffered saline (PBS, pH=7.4) for 2 hours, and post-fixed with 1% osmium tetroxide in the same buffer for 2 *hr*. After dehydration in an ascending series of ethanol, specimens were placed in acetone and embedded in epoxy resin. Thin sections were stained with uranyl acetate and lead citrate then examined using a transmission electron microscope (Zeiss, 911, Germany).

### TUNEL assay:

The human ovarian tissues and apoptosis-induced mouse thymus tissue were fixed in 4% formaldehyde and paraffin, serially cut into 5 *μm* sections and five sections were selected randomly for TUNEL assay (n=3 for each subgroup). The sections were stained according to the instructions of the kit (In Situ Cell Death Detection Kit, Roche, Germany). The slides were rinsed in PBS three times and examined using a fluorescence microscope (Zeiss, Axiophot, Germany). The photographs of each section were prepared and imported into Image J software (National Institutes of Health, Bethesda, USA), and the TUNEL positive signals per 1000 *μm*^2^ of ovarian tissue in three sections from each experimental subgroups were counted.

### Evaluation of apoptosis using DNA laddering:

After 14 days of culture, the human ovarian tissues and apoptosis-induced tissues were assessed for DNA laddering (n=3 in each subgroup). Total DNA was extracted from the tissues separately, according to the instruction of the kit (Apoptotic DNA Ladder Kit 1 835 246 Roche, Germany). The DNA content was determined spectrophotometrically. To assay DNA fragmentation, each lane of a 1% agarose gel was loaded with 10 *μg* DNA and electrophoresed for 45 *min* at 80 volts.

### Caspase-3/7 assay:

The caspase-3/7 activity of tissues from all experimental groups and dexamethasone treated mouse thymus tissue (n≤6 for each subgroup) was analyzed using the Caspase-Glu 3/7 Assay kit (Promega, G8020, UK), following the manufacturer’s instructions. The tissue extracts were cleared by centrifugation at 13000 *rpm* for 15 *min* at 4*°C* and the supernatants were divided into equal volumes for the total protein and caspase-3/7 assays. Total protein concentrations were determined by the Bradford technique (Bio-Rad). Diluted (10 *μg/ml*) extract was mixed with Caspase-Glo® Reagent in test tubes and incubated for 1 *hr* at room temperature. Following this, samples were put in a Sirius single tube luminometer (Berthold Detection Systems GmbH, Germany) and the reading on the luminometer was recorded in RLU (Relative light units). The activity of caspase-3/7 per mg protein was determined as described previously (29).

### RNA extraction and cDNA synthesis:

Total RNA was extracted from the human ovarian tissue samples and apoptosis-induced human ovarian tissues (n=3 in each subgroup) using an RNeasy Mini Kit (Qiagen, Valencia, CA, USA), according to the manufacturer’s instructions. Using oligo dT, RNA was reverse-transcribed by RevertAid M-MuLV reverse transcriptase using the specified primers (Table 1); the GAPDH gene was used as an internal control (26).

**Table 1.** Oligonucleotide primers

**Accession numbers**	**Gene**	**Primer sequence**	**PCR product size (*bp*)**
**NC_000012.11**	GAPDH	Forward:5′CTGGGCTACACTGAGCACC 3′ Reverse:5′AAGTGGTCGTTGAGGGCAATG3′	101
**NC_000010.10**	Fas	Forward: 5′TGAAGGACATGGCTTAGAAGTG 3′ Reverse:5′GGTGCAAGGGTCACAGTGTT3′	118
**NC_000001.10**	FasL	Forward: 5′GCAGCCCTTCAATTACCCAT 3′ Reverse:5′CAGAGGTTGGACAGGGAAGAA3′	101
**NC_000018.9**	Bcl2	Forward:5′TTGCTTTACGTGGCCTGTTTC3′ Reverse:5′GAAGACCCTGAAGGACAGCCAT3′	94
**NC_000019.9**	Bax	Forward: 5′CCCGAGAGGTCTTTTTCCGAG3′ Reverse:5′CCAGCCCATGATGGTTCTGAT3′	155
**NC_000017.10**	p53	Forward: 5′GAGGTTGGCTCTGACTGTACC3′ Reverse:5′TCCGTCCCAGTAGATTACCAC3′	133
**NC_000017.10**	BIRC5	Forward:5′AGGACCACCGCATCTCTACAT3′ Reverse:5′AAGTCTGGCTCGTTCTCAGTG 3′	118
**NC_000002.11**	caspase8	Forward: 5′ATTTGCCTGTATGCCCGAGC 3′ Reverse:5′CCTGAGTGAGTCTGATCCACAC3′	105
**NC_000004.11**	caspase3	Forward: 5′AGAGGGGATCGTTGTAGAAGTC 3′ Reverse:5′ACAGTCCAGTTCTGTACCACG3′	81

### Real time RT-PCR:

After cDNA synthesis, real time RT-PCR was performed on an Applied Bio-system real time thermal cycler using a Quanti-Tect SYBR Green RT-PCR kit (Applied Biosystems, UK).

After completing the PCR run, melt curve analysis was used to confirm the amplified product. For each sample, the reference gene (GAPDH) and the target genes were amplified in the same run. The real time thermal conditions included a holding step: 95°*C*, 5′, cycling step: 95°*C* 15′, 58°*C* 30′, 72°*C* 30′, and the cycle was continued by a melt curve step: 95°*C* 15′, 60°*C* 1′, 95°*C* 15′. Relative quantification of target genes was determined using the Pfaffl method (30). The real time RT-PCR experiments were repeated three times.

### Statistical analysis:

Quantitative variables were expressed as mean±SE. One-way ANOVA was used to compare the proportion of follicles among groups. Mean hormone levels were compared using independent t-tests and the day 2 and day 14 in each groups were compared with paired t-test. The results of the real time RT-PCR were compared by one-way ANOVA and the post hoc Tukey test. The p-values of <0.05 were considered significant.

## Results

**Figure 1.**
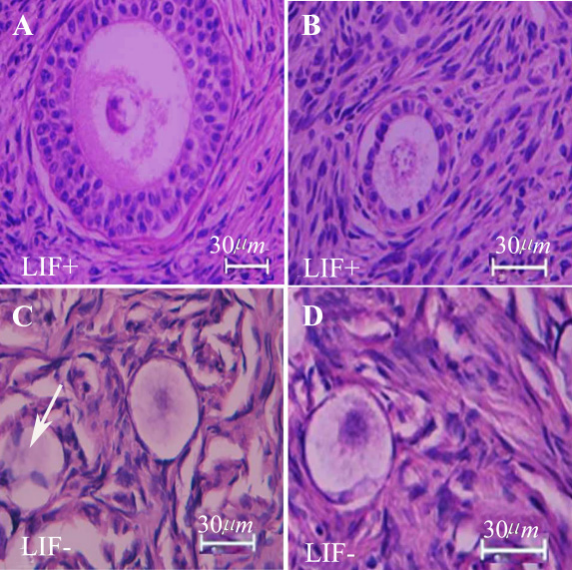
Light microscopy of human ovarian cortex after 14 days of *in vitro* culture; samples stained by haematoxylin and eosin. The morphology of follicles in the non-vitrified–LIF^+^ group (A), vitrified–LIF^+^ (B) non-vitrified–LIF^−^ (C) and vitrified–LIF^−^ (D) groups is shown. Damaged follicle with disorganized granulosa cells was seen (Arrow head)

The morphology of cultured human ovarian tissues in the vitrified and non-vitrified groups in the presence and absence of LIF, using H&E staining, is shown in figure 1A–D. The light microscopic observations showed the normal structure of follicles in cultured ovarian tissue. Some damaged follicles with disorganized granulosa cells were found in LIF non-treated group (Figure 1C, white arrow).

The percentage of normal follicles at different developmental stages in all groups is shown in table 2. There was a significant difference in the proportion of normal follicles between the nonvitrified–LIF^−^ and vitrified–LIF^−^ groups (p<0.05) but no significant difference was observed between the vitrified–LIF^+^ and non-vitrified–LIF^+^groups. The rates of normal follicles were higher in both LIF-treated groups than in non-LIF treated group (p<0.05). There was no significant difference in the proportions of primordial, primary and growing follicles between the non-vitrified and vitrified groups but these rates differed significantly in the LIF-supplemented groups in comparison with non-LIF treated group (p<0.05).

**Table 2.** The number and percentage of normal follicles at different developmental stages in all experimental groups

**Groups**	**Normal F.**	**Degenerated F.**	**Primordial F.**	**Primary F.**	**Growing F.**
	**(%±SE)**	**(%±SE)**	**(%±SE)**	**(%±SE)**	**(%±SE)**
**Non-vitrified- LIF ^−^**	27 (76±0.5)	9 (24±0.2)	21 (79±3.5)	5 (17.4±0.4)	1 (3.6±0.13)
**Vitrified-LIF ^−^**	42 (67.27±4.2) ^a^	18 (32.73±0.14) ^a^	42 (77.9±4.14)	10 (18.6±0.5)	3 (3.5±0.15)
**Non-vitrified -LIF^+^**	52 (88.67±5.6) ^b^	8 (13.3±0.15) ^b^	35 (65.23±3.02) ^b^	13 (24.31±0.3) ^b^	6 (10.46±0.17) ^b^
**Vitrified- LIF^+^**	43 (86.3±3.6) ^b^	7 (14±0.11) ^b^	28 (64.54±4) ^b^	11 (25.3±3.5) ^b^	5 (10.16±0.17) ^b^

All experiments were done at least in 5 repeats and n=5 in each group.

a: Significant differences with the same non-vitrified groups (p<0.05)

b: Significant differences with the same non-LIF treated-cultured groups (p<0.05)

### Hormonal assay:

The levels of hormones were significantly changed in all groups of study on day 14 than day 2 of culture period (Table 3). In all LIF-treated groups, the levels of 17-β estradiol and progesterone were higher and the level of DHEA was lower than non-LIF treated groups (p<0.05) at the end of culture period. However, there was no significant difference between vitrified and non-vitrified groups.

**Table 3.** The Level of 17-β estradiol, progesterone and dehydroepiandrostrone in all cultured groups at days 2 and 14 of cultivation period

**Groups**	**17-β estradiol (*pg/ml*) (Mean±SE)**		**Progesterone (*ng/ml*) (Mean±SE)**		**DHEA (*μg/ml*) (Mean±SE)**	

	**Day 2**	**Day 14**	**Day 2**	**Day 14**	**Day 2**	**Day 14**
**Non-vitrified-LIF^−^**	5725±278	8548±211^*^	63±4	83±2^*^	184±11	169±6^*^
**Vitrified-LIF^−^**	6100±656	7899±856^*^	60±5	75±3^*^	176±8	158±7^*^
**Non-vitrified-LIF**^**+**^	5829±643	28062±1003 ^a*^	72±8	251±12 ^a*^	186±15	94±3 ^a*^
**Vitrified-LIF**^**+**^	5200±223	27651±587 ^a*^	68±6	245±15 ^a*^	170±7	98±5 ^a*^

All experiments were done at least in 3 repeats and n=3 in each group.

a: Significant differences with the same non-LIF treated-cultured groups (p<0.05).

*: Significant differences between day 14 and day 2 in the same group (p<0.05)

### Ultrastructural observations:

**Figure 2.**
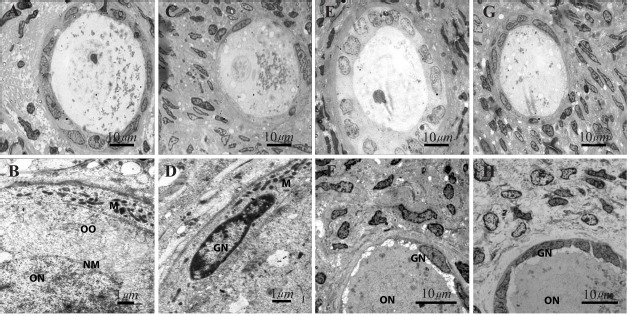
Ultrastructure of human ovarian follicles after 14 days of *in vitro* culture in the presence and absence of LIF. (A) Nonvitrified–LIF^+^ group; (B) Vitrified–LIF^+^; (C) Non-vitrified–LIF^−^; (D) Ditrified–LIF^−^; (E–H) High magnification images of previous groups, respectively. The chromatin in oocytes and follicular cells was normal in all groups. ON: Oocyte nucleus; NM: Oocyte nuclear membrane; OO: Ooplasm; GN: Granulosa cell nucleus; M: Mitochondria

The ultrastructure of human ovarian tissue at the end of cultivation period is shown in figure 2. The fine structure of all human ovarian culture groups was similar. The ultrastructure of normal follicles was seen in all groups of study including no chromatin condensation, shrinkage of cell membrane and cytoplasmic vacuoles. The oocytes had a euchromatin nucleus at the germinal vesicle stage and the granulosa and theca cell nuclei showed peripheral aggregations of heterochromatin.

### TUNEL assay:

**Figure 3.**
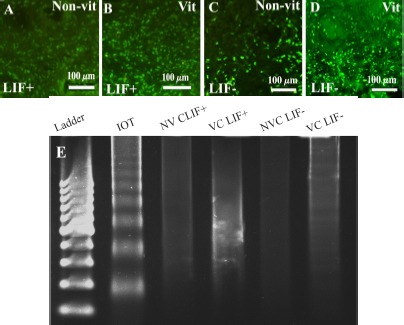
TUNEL assay in the non-vitrified–LIF^+^ group (A), vitrified–LIF^+^ (B) non-vitrified–LIF^−^ (C) and vitrified–LIF^−^(D) groups is shown. Total DNA gel electrophoresis in cultured human ovarian tissue (E). The DNA laddering pattern was present in the apoptosis-induced ovarian tissue (IOT) but not in the non-vitrified–LIF^+^ group (NVC LIF^+^), vitrified– LIF^+^ (VC LIF^+^), non-vitrified–LIF^−^ (NVC LIF^−^) and vitrified–LIF^−^ (VC LIF^−^) groups. Ladder: 100-bp molecular weight marker. The comparison of caspase 3/7 activity among all cultured human ovarian tissue is shown in part (B). Maximal activity was seen in apoptosis-induced mouse thymus (T). Non-vitrified–LIF^−^ (NVCLIF^−^); vitrified–LIF^−^ (VCLIF^−^); non-vitrified–LIF^+^ (NVCLIF^+^); vitrified–LIF^+^ (VCLIF^+^) groups. RLU: Relative luminescence *units/μg* of total protein. ^*^: Significant differences between LIF-treated and non-LIF-treated groups (p<0.05)

The TUNEL-positive cells were found in stromal tissue of cultured ovaries but the TUNEL signals were not seen in follicles of studied groups (Figure 3A–D). TUNEL signals per 1000 *μm*^2^ of ovarian tissue in the non-vitrified–LIF^−^, vitrified–LIF^−^, non-vitrified–LIF^+^ and vitrified–LIF^+^ groups were 5.22±0.23%, 6.21±0.25%, 2.14±0.19% and 2.39±0.22% per 1000 *μm*^2^, respectively. The number of apoptotic signals in vitrified samples was not significantly different from that in non-vitrified samples, but it was significantly lower in all LIF-treated groups than other groups (p<0.05). Mouse thymus tissue, used as a positive control, showed a high number of TUNEL signals per 1000 *μm*^2^ (8.62±0.32).

### DNA laddering:

Gel electrophoresis of genomic DNA from the apoptosis induced control human ovarian tissue showed characteristic pattern of DNA laddering (Figure 3E). Moreover, no DNA laddering pattern was observed on gel electrophoresis of all cultured groups (Figure 3E).

### Caspase-3/7 assay:

A high level of caspase-3/7 activity was observed in apoptosis-induced mouse thymus, used as the positive control. This enzyme activity was significantly lower in both LIF-treated groups than their control groups (p<0.05), but there were no significant differences between the non-vitrified and vitrified groups in this regard (Table 4).

**Table 4.** Caspase 3/7 activity in studied groups (mean±SE)

**Groups**	**Caspase 3/7 (*RLU/mg protein*)**
**Apoptosis induced mouse tymuse**	16370±658.49
**Non-vitrified-LIF^−^**	13533.33±785.98
**Vitrified-LIF^−^**	12700±351.11
**Non-vitrified-LIF^+^**	2158±34.034
**Vitrified-LIF^+^**	2042±70.868

There was no significant difference between groups. All experiments were done at least in 3 repeats and n=3 in each group

### Expression of apoptosis-related genes in cultured human ovarian tissue:

The ratio expression of proapoptotic (Bax, p53, Fas, FasL, caspase3 and 8) and anti-apoptotic (Bcl-2, BIRC5) genes to housekeeping gene (GAPDH) in all groups is shown in table 5 and table 6, respectively. Among the studied genes, the expression of Bcl-2, Bax, Bax/Bcl-2, caspase8 and BIRC5 was not significantly different in the non-vitrified and vitrified groups, but the expression of caspase3, p53, Fas and FasL was significantly higher in the absence of LIF in the vitrified group in comparison with the nonvitrified group (p<0.05).

**Table 5.** The expression of pro-apoptotic genes expression to housekeeping gene (GAPDH) in studied groups (Mean±SE)

**Groups**	**Fas**	**FasL**	**Bax**	**P53**	**Caspase3**	**Caspase8**
**Non-vitrified-LIF^−^**	0.0089±0.0004	0.0173±0.002	0.0007±0.0019	0.0142±0.0017	0.0021±0.0019	0.0957±0.004
**Vitrified-LIF^−^**	0.0215±0.0012 ^a^	0.0245±0.001^a^	0.0054±0.0032	0.3103±0.0019 ^a^	0.0052±0.0003 ^a^	0.1062±0.006
**Non-vitrified-LIF**^**+**^	0.0040±0.0067^b^	0.0086±0.0003 ^b^	0.0037±0.0004 ^b^	0.0062±0.0017 ^b^	0.0008±0.00009 ^b^	0.4620±0.002 ^b^
**Vitrified-LIF**^**+**^	0.0062±0.0001 ^a, b^	0.0062±0.0001 ^a,b^	0.0044±0.0005	0.0082±0.0004 ^a,b^	0.0006±0.0001^a,b^	0.0567±0.002^a,b^

a: Significant difference with non-vitrified group in respected group (p<0.05).

b: Significant difference with non-LIF-treated group in respected group (p<0.05).

All experiments were done at least in 3 repeats and n=3 in each group

**Table 6.** The expression of anti-apoptotic genes expression to housekeeping gene (GAPDH) in studied groups (Mean±SE)

**Groups**	**Bcl2**	**BIRC5**	**Bax/Bcl2**
**Non-vitrified-LIF^−^**	0.085±0.007	0.166±0.018	0.086±0.007
**Vitrified-LIF^−^**	0.073±0.005	0.149±0.018	0.074±0.011
**Non-vitrified-LIF**^**+**^	0.092±0.005	0.301±0.028 ^a^	0.039±0.003
**Vitrified-LIF**^**+**^	0.877±0.004	0.284±0.015 ^a^	0.049±0.012

a: Significant difference with non-LIF-treated group in respected group (p<0.05). All experiments were done at least in 3 repeats and n=3 in each group

In both LIF-treated (Vitrified and non-vitrified) groups, the expression ratio of pro-apoptotic genes (Fas, FasL, caspase 3 and 8, and p53) was lower and the expression of anti-apoptotic gene (BIRC5) was higher than non-LIF-treated groups (p<0.05).

## Discussion

Our observations showed a similar percentage of morphologically normal follicles in non-vitrified and vitrified groups and demonstrated that this vitrification technique had no significant effect on the viability of human ovarian follicles in long-term culture. Therefore, this method may allow preservation of the fine structure and integrity of different cells in ovarian tissue.

It was reported previously that the morphology of the human ovarian tissues was well preserved after short-term culture (25), and similar observations have been made by others (24, 28). However, some cryodamage to oocytes and follicular cells has been shown in vitrified human ovarian tissue (22, 31, 32, 33). These differences may be related to the cryopreservation protocols used, the type of cryoprotectants, and other individual differences between human samples. In addition, our biochemical analysis of apoptosis, including caspase-3/7 and TUNEL assays, showed no increase in the incidence of apoptosis in the vitrified groups during the culture period. However, apoptotic cells within the stroma of ovarian tissue may represent migratory cells around the blood vessels (25) or be related to vascular endothelial cells, connective tissue cells and corpora albicans in the ovarian tissue (34).

Our data showed that LIF improved the proportion of normal follicles (p<0.05); this is the first report regarding the effect of LIF on the improvement of follicular survival and development after *in vitro* culture of human ovarian tissue. In addition, an increase in the proportion of growing follicles and the well preserved ultrastructure of tissue in the LIF-treated groups showed its beneficial effects on preservation of follicles in human ovarian tissues during the culture period. LIF acts as a survival and proliferation factor in several cell types via phosphoinositide 3-kinase pathways (12, 13). It may also be involved in growth initiation of human primordial follicles, acting through its receptors, which are expressed on oocytes and granulosa cells in the primary and secondary follicles of adult human ovaries (35). Similar effects on the survival and growth of follicles have been shown in other species (20, 36–38).

Fewer TUNEL signals and a low level of caspase-3/7 in LIF-treated groups, shown for the first time in this study, demonstrated that LIF may support follicular survival by decreasing the incidence of apoptosis. Similar anti-apoptotic effects of LIF on different cell types, such as embryos, stem cells and myoblasts have been shown (13, 15, 39).

Our molecular observations, showed no significant change in the expression of most pro- and anti-apoptotic genes in vitrified and non-vitrified groups, but the expression of Fas and FasL in the vitrified groups was significantly higher than the one in the non-vitrified groups. However, in our previous study, it was shown that, immediately after warming in vitrified human tissue, there was no remarkable change in the expression of apoptosis-related genes (26). It seems that some alteration in the pattern of gene expression in vitrified samples needs more time to exhibit its effect, and therefore it is not detectable just after warming but detectable after 14 days of culture.

It has been suggested that the viability and follicular development may be affected by a high level of expression of pro-apoptotic genes, which leads to cell death within ovarian tissue; more studies at the protein level are need to confirm this hypothesis. Fas and FasL induce apoptosis through activation of caspase 3 and 8 (40), and some reports have shown that FasL is capable of inducing cell apoptosis (41).

However, the level of pro-apoptotic gene expression was significantly lower and the expression ratios of anti-apoptotic genes and Bax/Bcl-2 were significantly higher in all LIF-treated groups than the non-LIF treated groups. These data suggest that LIF may support and improve the viability of cultured ovarian tissue by decreasing the level of pro-apoptotic gene expression.

Bcl-2 is expressed in granulosa cells of both fetal and adult ovaries. It has a critical role in inhibiting the granulosa cell apoptosis pathway (42). Depalo et al. demonstrated that there is a significant positive correlation between Bcl-2 and the absence of apoptosis (43). Bax is a pro-apoptotic protein which is involved in granulosa cell apoptosis (42). The ratio of Bcl-2 to Bax within the cell controls the susceptibility of that cell to any given apoptotic stimulus (4, 5). In addition, the low level of p53 gene expression in the LIF-treated groups is an index of genome integrity and hence of the functional viability of the follicle. It has been shown that p53 protein was present in apoptotic cell nuclei of atretic follicles in rat ovaries (6).

## Conclusion

During *in vitro* culture of vitrified human ovarian tissue, the increasing level of E2 and P4 steroid hormones and decline in DHEA levels demon strated that the endocrine function of ovarian tissue is related to growing follicles and stromal cells especially in LIF treated groups.

Therefore, vitrification of human ovarian tissue did not increase the incidence of apoptosis at the morphological and molecular levels and that LIF may improve the survival and development of cultured follicles by decreasing the incidence of apoptosis and the expression of pro-apoptotic genes.
